# A New Behavioral Paradigm for Frustrative Nonreward in Juvenile Mice

**DOI:** 10.1016/j.bpsgos.2023.09.007

**Published:** 2023-11-17

**Authors:** Aijaz Ahmad Naik, Xiaoyu Ma, Maxime Munyeshyaka, Ellen Leibenluft, Zheng Li

**Affiliations:** a Section on Synapse Development Plasticity, National Institute of Mental Health, National Institutes of Health, Bethesda, Maryland; b Center on Compulsive Behaviors, Intramural Research Program, National Institutes of Health, Bethesda, Maryland; c Section on Mood Dysregulation and Neuroscience, National Institute of Mental Health, National Institutes of Health, Bethesda, Maryland

**Keywords:** Aggression, DMDD, Frustrative nonreward, Hyperlocomotion, Irritability

## Abstract

**Background:**

Irritability, defined as proneness to anger, can reach a pathological extent. It is a defining symptom of disruptive mood dysregulation disorder and one of the most common reasons youths present for psychiatric evaluation and care. Aberrant responses to frustrative nonreward (FNR), the response to omission of expected reward, are central to the pathophysiology of irritability. FNR is a translational construct to study irritability across species. The development of preclinical FNR models would advance mechanistic studies of the important and relatively understudied clinical phenomenon of irritability.

**Methods:**

We used FNR as a conceptual framework to develop a novel mouse behavioral paradigm named alternate poking reward omission. Juvenile mice were exposed to alternate poking reward omission and then examined with a battery of behavioral tests to determine the behavioral effect of FNR.

**Results:**

FNR increased locomotion and aggression regardless of sex. These behavioral changes elicited by FNR resemble the symptoms observed in youth with severe irritability. FNR had no effect on anxiety-like, depression-like, or nonaggressive social behaviors.

**Conclusions:**

Our alternate poking reward omission paradigm effectively elevated aggression and locomotion in juvenile mice. These frustration effects are directly related to behavioral symptoms of youth with severe irritability. Our novel behavioral paradigm lays the groundwork for further mechanistic studies of frustration and irritability in rodents.

Irritability, defined as proneness to anger, can reach a pathological extent and is one of the most common reasons for psychiatric evaluation and treatment in youth (1). It is a hallmark symptom of disruptive mood dysregulation disorder (DMDD), a diagnosis introduced into the DSM-5 in 2013. Irritability is associated with long-lasting adverse outcomes including high rates of school suspensions, hospitalizations, suicidality, and diagnoses of anxiety and depression (2,3). Current treatments for irritability are limited and not specific (4). Increased understanding of its neurobiological mechanisms is warranted to facilitate the development of novel specific interventions. However, despite increased irritability research over the past decade, the neuroscience of irritability is still in its infancy (5, 6, 7, 8). Progress has been slow partly due to the lack of behavioral paradigms for studying irritability in model organisms.

Youth with severe irritability have elevated responses to frustrative nonreward (FNR), i.e., the emotional and behavioral response to the omission of an expected reward (9). FNR is a translational cross-species construct that can be leveraged to uncover pathophysiological mechanisms of irritability. American psychologist Abram Amsel, who pioneered the study of FNR, proposed that instrumental behavior is learned through frustrating, rewarding, and punishing events (10, 11, 12, 13). In contrast to the rewarding and punishing events, little research has focused on the neural substrates of frustrating events. Amsel studied the effect of FNR on behavior using a double-runway setup in which rats were trained to pass 2 runways connected in a series to receive rewards at the end of each runway. After rats learned the task, FNR was introduced by reward omission (10,13). Amsel demonstrated that FNR invigorated behavior as indicated by faster running and increased resistance to extinction.

The double-runway paradigm, an operant conditioning chamber, has also been used to produce frustration in rodents. This method works better for adult animals with well-developed muscles than for juveniles because animals must press a lever repeatedly to receive reward. Both the double-runway and operant conditioning chamber methods require weeks of training (14, 15, 16). Long training periods are unsuited to studying irritability-like behavior in mice because DMDD is a disorder diagnosed in children of ages 6 to 18 years, which corresponds to 3 to 7 weeks of age in mice (17). The short developmental period of mice restrains the duration of the behavioral paradigm that can be used.

Human studies of brain-based mechanisms of irritability are limited and have yielded mixed results (18). Several studies have found circuitry abnormalities that show associations with irritability even after co-occurring symptoms of anxiety or attention-deficit/hyperactivity disorder are controlled (9,19). Developmental changes in the circuitry that mediates irritability or frustration have received little study, although one study (19) found the most marked aberrations in younger participants with relatively high levels of irritability. Recent network-based pilot studies using both task-based and resting-state functional magnetic resonance imaging found predictive associations between irritability and postfrustration connectivity in limbic, reward, and sensorimotor networks (9,20). However, the existing human neuroimaging literature does not allow for firm conclusions to be drawn about the circuitry that mediates irritability or a comparison between irritability and other related psychiatric disorders at the circuit level, emphasizing the need for animal models. The capacity to study neural mechanisms of irritability in animals would be a significant translational advance that could eventually lead to more precisely tailored innovative treatments for irritability and DMDD.

To study FNR in young mice, we developed a new behavioral paradigm named alternate poking reward omission (APRO). We show that after exposing juvenile mice to FNR using APRO, mice increase locomotion, aggression, and resistance to extinction of instrumental behavior.

## Methods and Materials

### Animals

All animal procedures followed the U.S. National Institutes of Health guidelines for using animals in intramural research and were approved by the National Institute of Mental Health Animal Care and Use Committee. C57BL/6J mice and BALB/c mice used in this study were bred in-house using breeders purchased from The Jackson Laboratory (#000664, #000651). Mice used in behavioral experiments were maintained under a 12-hour reversed light (8 pm–8 am)/dark (8 am–8 pm) cycle with access to water and food ad libitum except during the APRO period, when animals were given ad libitum access to water for 1 hour/day. Mice were individually housed for 2 days before aggression testing. For all other behavioral tests, 2 mice were housed in a divided cage and separated by a partition. Animals were randomly assigned to experimental groups.

### Alternate Poking Reward Omission

Mice (35 days old; male and female, *N* = 48; both control and FNR mice) corresponding to adolescence in humans were placed under water restriction for 3 days when they were given ad libitum access to water for 1 hour/day. The apparatus used for APRO was constructed by the National Institute of Mental Health Section on Instrumentation. It is a closed running track (50 cm long, 10 cm wide, 15 cm high) with a spout installed at each end and a light-emitting diode light mounted next to the spout. The mouse was allowed to run freely in the track for one 15-minute session per day. The mouse could poke the port when it reached the end of the track. If the mouse poked the port other than the previous one it had poked, the light-emitting diode light came on for 2 seconds, and a drop of water was delivered from the spout.

During training sessions, all mice received water reward for every correct alteration of port poking. Only mice making ≥12 licks on each side by day 3 proceeded to days 4 and 5 of APRO. On days 4 and 5, mice ran on the same track with the same rules. While control mice (*n* = 22) received water reward for all correct pokings on days 4 and 5, water reward was delivered for only 50% of correct pokings on day 4 and 20% of correct pokings on day 5 for mice exposed to FNR (*n* = 26). Thirty minutes after the FNR session on day 5, the mice were exposed to a battery of behavioral tests, including the open field, resident intruder (RI), elevated zero maze, light/dark box, forced swim, sucrose preference, and 3-chamber social preference tests. Different cohorts of mice were used for each behavioral test so that each mouse was exposed to only one behavioral test. For the extinction experiment performed on day 6, mice ran in the track for 15 minutes without light or water reward regardless of which port they poked. All behavioral tests were conducted under red lights during the dark cycle of the animal. Behavioral data were analyzed blindly.

### Behavioral Testing

#### Open Field Test

Mice were allowed to freely explore an opaque box (48 cm long, 48 cm wide, 40 cm high) for 20 minutes. The behavioral data were analyzed by using ANY-maze software (Stoelting) to calculate the total distance traveled and the time spent in the center zone that accounted for 25% of the total area.

#### RI Test

After the training session on day 3, mice were housed singly in cages. RI tests were conducted in the animal’s home cage. On day 5, mice were returned to their home cages after being tested on the linear track. A smaller BALB/c mouse of the same sex was introduced into the cage 10 to 15 minutes later. The cage was covered with a transparent plexiglass lid. The 2 mice were allowed to interact for 5 minutes. If bodily injury occurred due to the interaction between the resident and intruder mice, testing was discontinued, and the data were excluded from further analysis. Data from the RI test were analyzed manually and blind to experimental conditions. Based on previous studies, clinching, chasing, boxing, keeping down, and wrestling were identified as aggressive behavior (21,22). Anogenital sniffing, grooming, and inquiry were classified as nonaggressive social behaviors (23).

#### Elevated Zero Maze Test

Mice were allowed to freely explore an elevated zero maze (blue acrylic annular platform, 105 cm in diameter and 5.5 cm wide, elevated 60 cm above the ground) for 5 minutes. The maze consisted of 2 closed quadrants with 30-cm-high walls and 2 open quadrants with no walls. ANY-maze software was used to calculate the amount of time spent in and entries into the open quadrant.

#### Light/Dark Box Test

The test box was divided into 2 compartments (27 cm long, 27 cm wide, and 30 cm high) with an opening in between to allow the mouse to enter. The light chamber had white walls and was illuminated, while the dark compartment had black walls and was not illuminated. Mice were placed in the light box when the test started and allowed to freely explore the box for 5 minutes. ANY-maze software was used to calculate the percentage of time spent in the light compartment and the number of transitions between the light and dark compartments.

#### Forced Swim Test

Mice were gently released into a plexiglass cylinder (20 cm high, 10 cm deep) filled with water (23 °C, 7.5 cm deep) and left in the water for 5 minutes. ANY-maze software was used to calculate the total immobility time, number of immobile episodes, and delay to the first immobile episode.

#### Three-Chamber Social Preference Test

The social preference test described by Rein *et al.* (24) was used with some modifications. The test had 3 phases: habituation, pretesting, and testing. During habituation, the openings between the central and side chambers were blocked. There was an empty metal wire cup inside each side chamber. The mouse was placed in the central chamber and allowed to freely explore for 5 minutes. During the pretest, blocks between the central and side chambers were removed. Identical objects (blue Lego bricks) were placed in the cups. The mouse was allowed to interact freely with the objects for 5 minutes. After the pretesting phase, the mouse was held temporarily in the central chamber. One object was replaced by a social stimulus (an age- and sex-matched C57BL/6 mouse), and another object was replaced by a novel object (hexagonal Lego brick). The mouse was then released to freely explore the social and nonsocial stimuli for 10 minutes. ANY-maze software was used to record the time spent with social stimuli (mouse) and a nonsocial stimulus (object) during the test session.

#### Sucrose Preference Test

The sucrose preference test was performed as described by Liu *et al.* (25), with modifications. The test has 3 phases: adaptation, baseline measurement, and testing after FNR. During the adaptation phase, 2 bottles, one containing water and the other one containing 2% sucrose solution, were inserted into the cage lid. The mouse (P29) was housed in a cage with 2 bottles for 2 days. The position of the bottles was switched on the second day. Both bottles were removed at 6 pm on the second day. The baseline measurement started at 9 am on the following day. Fresh bottles containing 2% sucrose and water were weighed and inserted into the cage lid. The bottles were weighed 6 hours and 22 hours later to determine the baseline sucrose preference index. Mice that consumed no sucrose solution during the baseline measurement period (1 of 17 mice) were excluded from further testing. The sucrose preference testing after FNR started 1 hour after the animal was returned to its home cage following the FNR session. A weighed water bottle and sucrose solution bottle were inserted into the cage lid. The bottles were weighed 6 hours and 22 hours later to determine the post-FNR sucrose preference index.

### Data and Statistical Analysis

GraphPad Prism (9.5.1) was used for statistical analysis. For behavioral analysis, two-tailed Student’s *t* tests were used for normally distributed data with equal variance, and Mann-Whitney *U* tests were used for data that did not satisfy these requirements. One-way analysis of variance on ranks or two-way analysis of variance with Dunn’s procedures or Tukey’s test for post hoc multiple comparisons were used for comparing more than 2 groups. *p* = .05 was considered significant. All data are presented as mean + SEM. Statistical results for all figures are provided in Table S1.

## Results

### A New Behavioral Paradigm Based on the Frustrative Nonreward Framework

**Figure 1:**
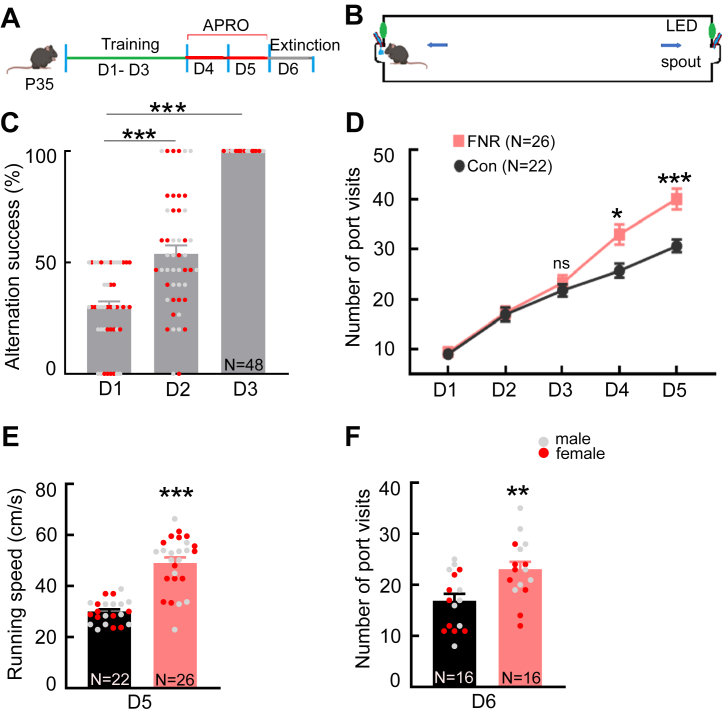
A novel frustrative nonreward paradigm applicable to juvenile mice. **(A)** Schematic of the experimental paradigm showing the 2 stages of APRO that begin on P35: training (D1–D3) and reward omission (frustrative nonreward D4 and D5). An extinction protocol was applied on D6 to test the frustrative nonreward effect. **(B)** Schematic drawing of the closed track apparatus installed with infrared-based sensors for poke-triggered cue light and reward delivery system. **(C)** The percentage of successful alternations between 2 ports across the 3 trainings days. **(D)** The number of port visits in the closed track across days. **(E)** The average instantaneous running speed between ports on D5. **(F)** The number of port visits during extinction on D6. Data are presented as mean + SEM; the numbers in the bars represent the number of animals. ∗*p* < .05, ∗∗*p* < .01, ∗∗∗*p* < .001. APRO, alternate poking reward omission; Con, control; D, day; FNR, frustrative nonreward; LED, light-emitting diode; ns, not significant; P35, postnatal day 35.

To study FNR, we designed a reward-seeking task that a juvenile mouse can learn within 3 days. We named this behavioral paradigm APRO. APRO, conducted in a linear track, had 2 stages that were completed in 5 days (Figure 1A, B). The rate of correct port poking increased over the training period and reached 100% on day 3 (Figure 1C and Table S1). For mice exposed to FNR (FNR mice), frustration was then induced by withholding reward 50% of the time on day 4 and 80% of the time on day 5.

FNR mice increased the number of visits to the ports on days 4 and 5 (Figure 1D and Table S1). In addition, FNR mice ran faster during the test session than control mice on day 5 (Figure 1E and Table S1). When testing extinction on day 6, FNR mice (*n* = 16, 8 female and 8 male) made more visits to the ports than control mice (*n* = 16, 8 female and 8 male) (Figure 1F and Table S1), indicating increased resistance to extinction.

Taken together, the behaviors of FNR mice in the APRO paradigm are consistent with Amsel’s proposition that frustration increases running speed and resistance to the extinction of instrumental behavior.

### FNR Increases Locomotion

**Figure 2:**
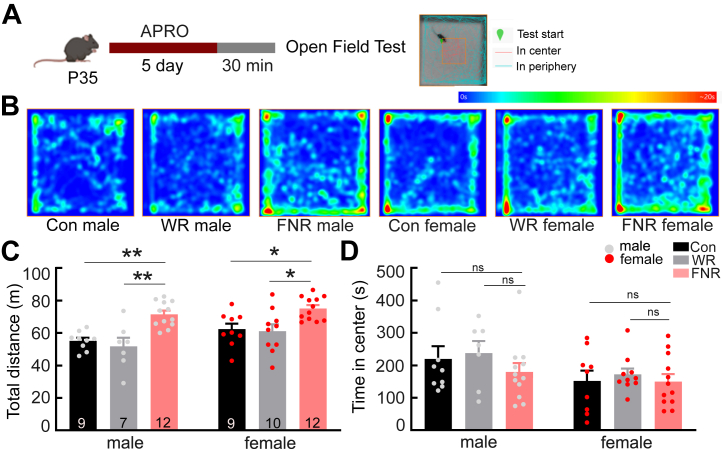
FNR induces hyperlocomotion in both male and female mice. **(A)** Experimental schedule of APRO followed by open field test and illustration of division of the central and peripheral zones; the cyan and red lines indicate the mouse’s trajectory in the testing arena. **(B)** Representative mobility heatmaps showing the activity of male and female mice from different groups within the open field arena. **(C)** Total distance traveled by control, water-restricted, and frustrated mice grouped by sex in a 20-minute testing session. **(D)** Total time spent in the central zone (25% of the testing arena) by male and female mice of different groups. Data are presented as mean + SEM; the numbers in the bars represent the number of animals. ∗*p* < .05, ∗∗*p* < .01. APRO, alternate poking reward omission; Con, control; FNR, frustrative nonreward; ns, not significant; P35, postnatal day 35; WR, water-restricted only.

We assessed the effect of FNR by conducting additional behavioral tests. Frustration is conceptualized as an adaptive motivational state that elevates activity and aggression (22,26, 27, 28, 29, 30). Therefore, we examined locomotion with the open field test (Figure 2A). Mice were tested within 15 minutes after the test session of APRO on day 5. FNR mice (*n* = 24, 12 female and 12 male) traveled a longer distance in the arena during the open field test than control mice (*n* = 18, 9 female and 9 male) (Figure 2B, C and Table S1). However, the time spent in the center zone of the arena, which is an indicator of anxiety-like behavior, was comparable in control and FNR mice (Figure 2B, D and Table S1). Both male and female FNR mice exhibited hyperlocomotion (Figure 2B, C and Table S1).

The mice were under water restriction throughout APRO. Because control mice drank more water than FNR mice in the track prior to the open field test, thirst may underlie the hyperlocomotion of FNR animals. To test the effect of thirst on locomotion, we conducted the open field test in mice that had been water restricted but not exposed to FNR (*n* = 17, 10 female and 7 male). Water-restricted mice and control mice were comparable in the total distance traveled and time spent in the center zone (Figure 2B–D and Table S1). These results indicate that FNR, but not thirst, increased locomotion.

### FNR Increases Aggression but Not Nonaggressive Social Interaction

**Figure 3:**
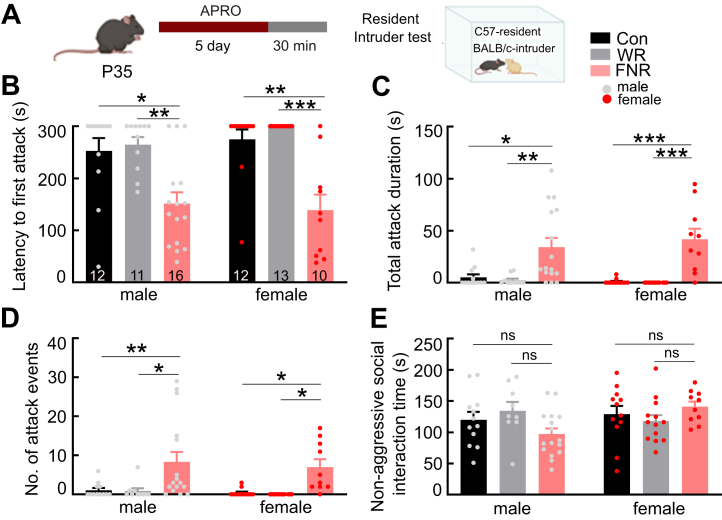
FNR increases aggressive behavior in both male and female mice. **(A)** Experimental schedule of APRO followed by the resident intruder test. **(B)** The latency to the first attack in male and female mice. **(C)** The total time the resident mouse spent attacking the intruder. **(D)** The number of attack events during the resident intruder test. **(E)** The total time spent in nonaggressive social interaction. Data are presented as mean + SEM; the numbers in the bars represent the number of animals. ∗*p* < .05, ∗∗*p* < .01, ∗∗∗*p* < .001. APRO, alternate poking reward omission; Con, control; FNR, frustrative nonreward; ns, not significant; P35, postnatal day 35; WR, water-restricted only.

Next, we examined the effect of FNR on aggression using a modified RI test (Figure 3A). An FNR or control mouse (resident) was placed back in the home cage after the test session on day 5 and allowed to freely explore the cage for 10 to 15 minutes. An unfamiliar, smaller mouse of the same sex (the intruder) was then introduced into the same cage to interact with the resident mouse for 5 minutes (Figure 3A).

The control mice largely displayed nonaggressive social behaviors such as anogenital sniffing, grooming, and rearing toward the intruder. However, the FNR mice exhibited more aggressive behaviors toward the intruder, such as clinch attack, keep down, lateral threat, and chasing (21,23,31). Compared with control mice (*n* = 24, 12 female and 12 male), FNR mice (*n* = 26, 10 female and 16 male) had a higher number of total attacks, a higher average attack duration per episode and total attack time, and a shorter latency to the first attack (Figure 3B–D and Table S1). Notably, C57BL/6 female mice that are usually not aggressive in the RI test also attacked the intruder after FNR (Figure 3B–D and Table S1). Water restriction alone had no effect on aggressive behavior (*n* = 24, 13 female and 11 male) (Figure 3B–D and Table S1). Nonaggressive social behavior during the RI test was comparable in FNR and control mice (Figure 3E and Table S1). Previous studies have shown that juvenile mice have low levels of aggression (32,33). This is consistent with our findings in control mice. However, FNR made juvenile mice much more aggressive, demonstrating the ability of the paradigm to elicit an important and predicted behavioral response.

**Figure 4:**
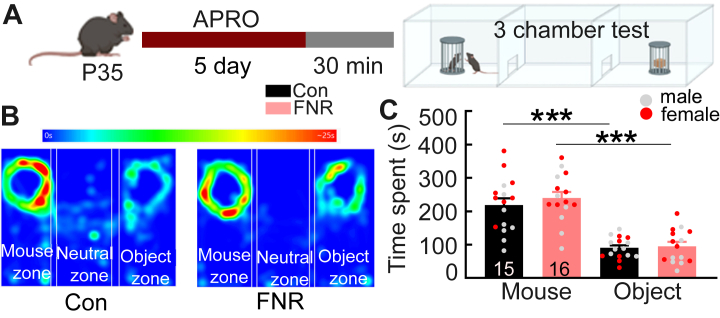
FNR has no effect on social preference. **(A)** Experimental schedule of APRO followed by the 3-chamber social preference test. **(B)** Representative heatmaps showing the activity of the mouse in the mouse and object zone along with the neutral zone. **(C)** The total time spent with social stimulus (another mouse) and nonsocial stimulus (object) during the test by control and FNR groups. Data are presented as mean + SEM. *n* = 16 for each group. ∗∗∗*p* < .001. APRO, alternate poking reward omission; Con, control; FNR, frustrative nonreward; P35, postnatal day 35.

We further assessed the effect of FNR on social behavior using the 3-chamber sociability test (24) (Figure 4A). FNR (*n* = 16, 8 female and 8 male) and control mice (*n* = 15, 8 female and 7 male) spent similar amounts of time exploring the conspecific and object (Figure 4B, C and Table S1), indicating comparable sociability. Males and females were pooled in data analysis because no sex differences were detected (Table S2). Taken together, these findings indicate that FNR elevated aggression in both male and female mice without altering nonaggressive social interaction or sociability.

### FNR Has No Effect on Anxiety-like or Depression-like Behavior

**Figure 5:**
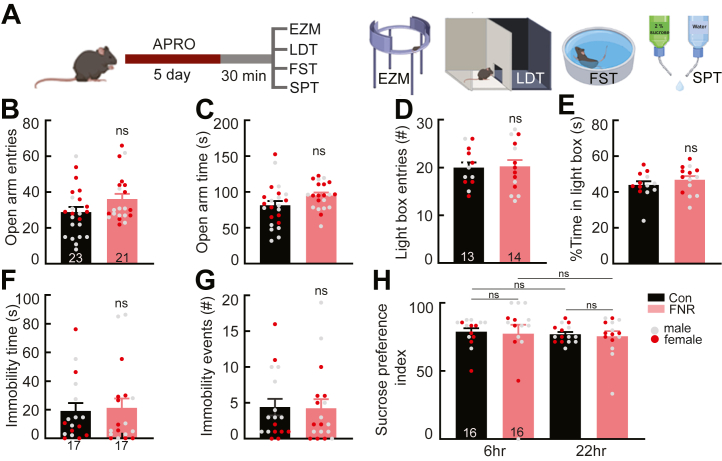
FNR has no effect on anxiety-like or depression-like behaviors. **(A)** Schematic drawing of the experimental schedule. The EZM, LDT, FST, and SPT followed APRO; different cohorts of mice were used for each test. **(B)** Total entries of the open arms in the EZM test. **(C)** Total time spent in the open arms during the EZM test. **(D)** Total entries into the light box in the light/dark box test. **(E)** Total time spent in the light box during the light/dark box test. **(F)** Total time the mouse was immobile during FST. **(G)** The number of immobile events during FST. **(H)** The ratio of sucrose consumption over water recorded 6 hours and 22 hours after the day 5 test session in control and FNR group mice is shown. Data are presented as mean + SEM; the numbers in the bars represent the number of animals. APRO, alternate poking reward omission; Con, control; EZM, elevated zero maze; FNR, frustrative nonreward; FST, forced swim test; LDT, light/dark box test; ns, not significant; SPT, sucrose preference test.

To test whether FNR affects anxiety-like behavior, we conducted the light/dark box and elevated zero maze tests after the test session on day 5 (Figure 5A). Each test used a different cohort of animals. In the elevated zero maze test, the time spent in open arms and the total number of entries into open arms were comparable in control (*n* = 23, 10 female and 13 male) and FNR (*n* = 21, 11 female and 10 male) (Figure 5B, C and Table S1) mice. Similarly, in the light/dark box test, the time spent in the light box and the total number of entries into the light box were comparable in control (*n* = 13, 7 female and 6 male) and FNR (*n* = 14, 7 female and 7 male) (Figure 5D, E and Table S1) mice. Male and female mice were pooled in data analysis because no sex differences were detected (Table S2). These results indicate that FNR had no effect on anxiety-like behavior.

For depression-like behavior, we used the forced swim and sucrose preference tests to assess behavioral despair and anhedonia-like behavior. In the forced swim test, total immobility time and total number of immobile episodes were comparable in control (*n* = 17, 8 female and 9 male) and FNR (*n* = 17, 8 female and 9 male) (Figure 5F, G and Table S1) mice. In the sucrose preference test, the ratio of sucrose to water consumed during the 6-hour and 22-hour periods either before or after APRO was comparable in control (*n* = 16, 8 female and 8 male) and FNR (*n* = 16, 8 female and 8 male) (Figure 5H and Tables S1 and S2) mice. Male and female mice were pooled in data analysis because no sex differences were detected (Table S2).

These results indicate that FNR had no effect on anxiety-like or depression-like behaviors.

## Discussion

Irritability is among the most common reasons that children are brought for psychiatric evaluation and care (1,2,6,34,35). Despite the significant impact of irritability on mental health, little is known about its underlying neural mechanisms, and limited treatment options are available (4,36). Mechanistic understanding of irritability is needed to guide the development of new treatments. Youths with severe irritability have elevated responses to FNR; the latter is a cross-species translational construct in the negative valence system of the Research Domain Criteria (9,36,37). Therefore, translational research elucidating the neurobiology of FNR can facilitate research on the pathophysiology of irritability. In addition, even though Amsel’s frustration theory has been examined extensively from a behavioral perspective since the 1950s, few studies have attempted to disentangle the underlying brain circuits and neural mechanisms.

We developed a mouse behavioral paradigm (APRO) based on FNR to study irritability-like behavior in juvenile mice. This paradigm takes advantage of the mouse’s natural tendency of alternating visits between 2 places to complete training within 3 days, significantly shorter than older paradigms, e.g., double runway and operant conditioning (14, 15, 16). We demonstrated that following APRO, FNR mice increased locomotion, aggression, and resistance to extinction of learned behavior. These behavioral alterations are consistent with frustration effects reported in rats, chimpanzees, pigeons, and humans (10,11,13,26, 27, 28, 29,38, 39, 40, 41, 42). However, our FNR mice showed no change in anxiety-like or depression-like behavior or social preference. These findings suggest that APRO induces relatively specific behavioral changes that are directly relevant to those seen in youths with severe irritability who have frequent temper outbursts characterized by increased motor activity and aggression (42,43). It is noted that APRO has more selective behavioral effects than frustration induced by extinction tasks that completely omit reward (44).

Our APRO paradigm produces robust and specific behavioral effects that recapitulate the behavioral phenotypes of DMDD. The full capacity of this novel paradigm in studying irritability has yet to be determined by more comprehensive future studies to address such questions as whether the frustration effect is strain specific, how long the frustration effect lasts, whether there are other behavioral domains that are also affected, whether the FNR effect is age dependent, and whether APRO is still effective in adults. In addition, while our experiments show that water restriction before APRO does not increase locomotion or aggression because control mice drank more water than FNR mice in the test session, whether or not the FNR effect is modified by the total amount of reward during the FNR session remains to be addressed by future studies. The normative developmental trajectory of irritability includes a peak during the preschool years followed by a gradual decline into adulthood. However, impairing irritability can show diverse trajectories (45). There is some evidence for comorbidity patterns that differ by sex and age, with irritability showing a particularly strong association with attention-deficit/hyperactivity disorder in young boys and with depression in adolescent girls (46), but more research is needed to understand how the comorbidity of pathological irritability changes over time. Irritability in youth is associated with increased risk for unipolar depression and anxiety (8).

It is possible that APRO produces strain-specific behavioral changes given the genetic, physiological, and behavioral variations among mouse strains. If this is true, research on strain-dependent responses to APRO would facilitate clinical research on individual differences in irritability. Applying APRO to mice predisposed to anxiety-like or depression-like behavior, such as mice harboring mutations in genes that increase risk for psychiatric disorders or mice exposed to environmental risk factors, is another avenue of future research that could provide insights into the complex pathogenic mechanisms of human irritability.

Despite limitations, our study provides a novel behavioral paradigm for studying FNR in juvenile mice. APRO can be used for interrogation of brain activity changes in response to FNR. APRO lays a foundation for further research that precisely manipulates and measures neural activity with advanced genetic, optogenetic, and electrophysiological tools to unveil the neural mechanisms of frustration and irritability.

## Supplementary Material

Supplementary Tables

Key Resources Table
